# Metal Particles and Extrapulmonary Transport: Oberdörster and Elder Respond

**Published:** 2007-02

**Authors:** Günter Oberdörster, Alison Elder

**Affiliations:** Department of Environmental Medicine, University of Rochester, Rochester, New York, E-mail: gunter_oberdorster@urmc.rochester.edu

We appreciate the opportunity to address the points raised by Ghio and Bennett in their letter. The pH of our cell-free dissolution studies (Elder et al. 2006) was appropriate because the nasal cavity surface is neutral and does not have airway macrophages and the phagolysosomal pH of nasal epithelial cells is neutral (Johnson 1994). Acidic pH, as in the phagolysosome of alveolar macrophages, dissolves manganese oxide, resulting in increased levels of blood-borne non-particulate Mn. Our neutral pH buffer dissolved only 1.5% of Mn oxide nanoparticles in 24 hr. The dissolution buffer was not “normal saline,” as stated by Ghio and Bennett; it was physiologic saline, a “simulated lung fluid” used for decades in studies of man-made fiber dissolution (Potter and Mattson 1991). It was an oversight on our part to omit the exact composition from our article (Elder et al. 2006), which includes citrate (model organic acid) and glycine (model protein component). Citrate is a stronger metal chelator than glycine.

If only soluble Mn translocates, the time required for solubilization would significantly retard the increase of Mn in the olfactory bulb of administered Mn oxide nanoparticles. Instead, we found no difference between the two forms of Mn when we directly compared the translocation rate of the Mn oxide nanoparticles to soluble Mn chloride (Elder et al. 2006), indicating a direct uptake of the Mn oxide nanoparticles by olfactory neuronal structures and subsequent translocation. It is conceivable that subsequent dissolution in the olfactory system occurs. The rapidity of solid nanoparticle transport along neuronal axons is, indeed, remarkable (~ 2.5 mm/hr), as demonstrated earlier by the arrival in the olfactory bulb of 50 nm gold particles within 30 min after intranasal instillation. This and other studies with gold nanoparticles using transmission electron microscopy detection (reviewed by Oberdörster et al. 2005) demonstrate unequivocally that some metal particles are indeed appropriate for demonstrating solid particle transport across epithelial barriers, refuting the absolute statement in the title of Ghio and Bennett’s letter.

Ghio and Bennett suggest that a carbon-based particle would be appropriate for studying ultrafine particle transport and translocation. This is true for elemental and organic carbon only if insoluble *in vivo*. Indeed, study with inhaled ultrafine elemental carbon particles (^13^C) confirmed their translocation to the olfactory bulb of rats (Oberdörster et al. 2004). In contrast, labeled elemental carbon is problematic, as pointed out by Ghio and Bennett regarding Technegas (^99m^Tc labeled-ultrafine carbon). Recent studies using inhaled Technegas (Mills et al. 2006; Wiebert et al. 2006) showed no translocation in humans, contradicting earlier work (Nemmar et al. 2002). Both leaching of the soluble radiolabel and the inability of the g-camera to detect small amounts (£ 1%) of the deposited dose in extrapulmonary organs are significant limitations with this noninvasive technique, resulting in misinterpretions suggesting either significant particle translocation or lack thereof.

Ghio and Bennett cite LeFevre et al. (1982), apparently as evidence that inhaled carbon-based particles do not undergo extrapulmonary transport. However, LeFevre et al. interpreted their findings of high black pigment scores in liver and spleen of coal miners differently—namely, as migration of coal dust in the pneumoconiotic lung into pulmonary lymphatics and then to the systemic circulation, and also as migration of coal mine dust–laden macrophages through the walls of pulmonary blood vessels. Whatever the mechanism, their findings clearly indicate extrapulmonary transport. Heavy silica inhalation exposure has also been found to result in particle accumulation in liver and other extrapulmonary tissues in humans and nonhuman primates (Carmichael et al. 1980; Rosenbruch 1990; Slavin et al. 1985). Ghio and Bennett further state that

Decades of research have provided no evidence of an extrapulmonary transport (including via olfactory neuronal pathways) of particles associated with cigarette smoking.

How many investigators have tried to find cigarette smoke particles in extrapulmonary tissues? We are aware of only one study in rats in which a 25-min inhalation exposure to ^14^C cigarette smoke resulted in 0.24–0.83% of retained ^14^C in the liver within 15 min after the short-term exposure (Chen et al. 1989). Does this indicate extrapulmonary particle transport? Possibly yes.

We conclude that the physicochemical characteristics of a nanoparticle—whether metal or not—and the physiologic milieu at the site of deposition in the respiratory tract determine whether extrapulmonary translocation occurs as particle or as solute. A prerequisite for noninvasively measuring this is that the detection method has sufficient sensitivity for identifying the analyte at expected low translocation rates.

Regarding our nasal translocation study in rats (Elder et al. 2006), we conclude that inhaled Mn oxide nanoparticles are taken up by the nasal olfactory neuronal pathway as solid particles rather than being slowly dissolved first at neutral pH. For the alveolar region where dissolution is expected to be more rapid, this does not apply.
